# Building on muscles: how built environment design impacts modern sports science

**DOI:** 10.1136/bmjsem-2024-001908

**Published:** 2024-03-14

**Authors:** Mohammad Javad Koohsari, Andrew T Kaczynski, Motohiko Miyachi, Koichiro Oka

**Affiliations:** 1 School of Advanced Science and Technology, Japan Advanced Institute of Science and Technology, Nomi, Japan; 2 Faculty of Sport Sciences, Waseda University, Tokorozawa, Japan; 3 School of Exercise and Nutrition Sciences, Deakin University, Geelong, VIC, Australia; 4 Department of Health Promotion Education and Behavior, Arnold School of Public Health, University of South Carolina, Columbia, SC, USA

**Keywords:** Environment, Sports, Performance, Athletics

## Abstract

Sports science focuses on enhancing athletes’ performance, requiring a multifaceted approach. It is evolving from a purely muscle-centred approach to an interdisciplinary one. This paper investigates built environment design science, a dimension less explored in relation to enhancing athlete performance in sports science. The discussion is divided into three categories: athlete-centric training built environment design, enhanced fan and community engagement, and improved integrative accessibility. The study also identifies future research directions, including evidence of the relative impact of the built environment, financial aspects, and performance evaluation methods. Collaboration between sports scientists and scholars in urban design, parks, transportation, landscape architecture and environmental psychology is necessary to advance this topic further.

**WHAT IS ALREADY KNOWN ON THIS TOPIC**

- The built environment is recognised for its impact on health behaviours, yet its specific influence on athletic performance in sports contexts remains underexplored in existing research.

**WHAT THIS STUDY ADDS**

- This study introduces how (re)designing built environments can positively affect athletic performance, focusing on athlete-centric training environments, community engagement and accessibility.
- It highlights the connection between sports science and built environment design science, emphasising an interdisciplinary approach.

**HOW THIS STUDY MIGHT AFFECT RESEARCH, PRACTICE OR POLICY**

- The insights from this study could encourage educational institutions to include built environment design subjects in sports science curricula.
- It can also influence urban design and sports facility development policymaking, encouraging a more athlete-friendly built environment design approach.

## Introduction

Over the past two decades, there has been a significant increase in scholarly interest in sports science. This trend is demonstrated by the notable growth in student enrolment and graduation rates from sports science programmes.^1 2^ Sports science can be defined as applying scientific principles to inform the practice of sports, aiming to improve athletes’ performance.^3^ It encompasses ‘the study of maximising competitive athletic performance’.^4^ These definitions underscore the discipline’s focus on optimising athletes’ performance. Improving such performance is a multifaceted challenge involving numerous variables such as physical training, mental focus, nutritional strategies and physiological resilience.^5^ The complexity of these variables requires an interdisciplinary approach to gain comprehensive insights and develop effective strategies for athlete enhancement.^6^

There is a growing interest in how (re)designing the built environment can shape human health behaviours. The built environment refers to ‘the human-made space in which people live, work and recreate on a day-to-day basis’.^7^ This includes all physical settings such as houses, shops, streets, parks, libraries, schools and stadiums. Many studies have investigated the influence of built environment design on physical activity,^8^ dietary intake^9^ and several health biomarkers.^10^ Nevertheless, current evidence primarily focuses on the general population. It is not yet clear how (re)designing the built environment may specifically enhance performance among athletes at various levels. Therefore, in this interdisciplinary paper, we discuss how (re)designing the spaces we inhabit may influence athletic performance.

## The built environment design science and athletes’ performance

**Figure 1:**
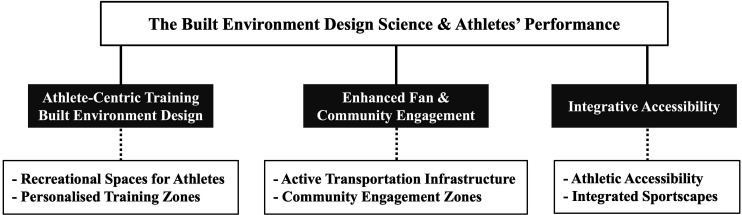
The built environment design science and athletes’ performance.

Athletes’ performance is affected by built environment attributes across various spatial settings, such as schools, stadiums, training arenas and athletic complexes.^11–13^ We categorise how built environment design influences athletes’ performance into three areas: athlete-centric training built environment design, enhanced fan and community engagement, and integrative accessibility (figure 1).

### Athlete-centric training built environment design

Moving beyond a conventional equipment-centric approach, built environment design can create an athlete-centric environment prioritising athletes’ needs in at least two ways:

#### Recreational spaces for athletes

Empirical evidence indicates a link between indoor built environment attributes and residents’ mental health.^14^ This link is partly established through exposure regulation to nature, shade and building air quality.^15^ Similarly, built environment design can integrate physical elements to improve athletes’ relaxation and mental health. For instance, designing training facilities with open-air lounges, ergonomic furniture and greenery may provide athletes with a dedicated space for recovery and restoration between workouts.

#### Personalised training zones

Built environment design can impact the creation of individualised environments through spatial arrangements for specific athlete types. This may include designing specialised recovery areas and spatial layouts to meet each athlete’s needs for optimal performance. For example, a training facility for swimmers and runners might feature an adjustable aquatic centre and a multi-functional indoor track. Customised recovery zones complement each area. However, no evidence exists on each sport type’s most effective spatial design.

### Enhanced fan and community engagement

Enhanced fan and community engagement can boost athletes’ performance by motivating them.^16^ The built environment design may act as a catalyst for this purpose through the following areas:

#### Active transportation infrastructure

Designing the built environment to include active transportation infrastructure near sports facilities can enhance fan and community engagement. Creating safe and accessible paths for cycling and walking that connect sports venues with urban areas may encourage active transportation among event attendees. For instance, a study during the FIFA Confederations Cup in Brazil in 2013 found fan walking choices were significantly influenced by built environment factors such as accessibility and pedestrian comfort.^17^ Likewise, accessible public transportation can mitigate physical inactivity and traffic congestion around stadiums. It can also help mitigate air and noise pollution in these highly frequented spots.^18^

#### Community engagement zones

Professional sports events can act as a catalyst for community development and capacity building.^19^ Designing vibrant plazas adjacent to sports facilities may directly promote community engagement. These spaces can also be equipped with interactive displays, community events, and athlete meet-and-greet sessions, fostering a supportive atmosphere for athletes.

### Integrative accessibility

At a more macro spatial scale, built environment design can influence athletes’ performance through enhanced accessibility and functional dimensions.

#### Athletic accessibility

The built environment design can strategically place sports facilities near health services such as medical clinics and physiotherapy centres. This proximity can enhance athletes’ access to recovery and injury prevention during training and competitions. Additionally, developing a network connecting various sports venues with efficient public transport options facilitates convenient commutes between training and competition venues.

#### Integrated sportscapes

With the rise of automobile use in the 1950s, stadiums moved to suburban areas, away from city centres and downtowns.^20^ These locations, often near highways, can intensify the sense of isolation for fans and athletes. Integrating sports facilities within the urban fabric, aligning them with the city’s aesthetics, may improve athletes’ sense of belonging and comfort. For instance, (re)developing sports arenas in downtown areas may stimulate local economic growth, housing values and fan attendance.^21–23^ The architectural design of these stadiums and facilities can also play a role in revitalising their surrounding neighbourhoods.^24^

## Conclusion and future directions

Athletes in many sports have reached near-peak performance, necessitating innovative ideas for further improvement in professional competitions.^25^ This paper highlighted the potential of built environment design science to enhance these performance levels. Several key areas emerge that require further investigation:

- *The relative impact of built environment design*. There is insufficient evidence on the extent of the built environment’s impact on athlete performance. Quantifying the contribution of built environment factors compared with other performance influencers is a key area for future research.
- *Financial and temporal requirements for built environment interventions*. Future research is needed to examine the financial and temporal investments necessary to develop and implement built environment facilities and infrastructure. Such studies need to clarify the feasibility of built environment initiatives.
- *Cost responsibility*. Future studies should explore allocating financial responsibilities among athletes, local governments and other stakeholders for built environment interventions.
- *Metrics for athlete performance evaluation*. In built environment and health research, outcomes are typically measured by risks such as death or disease. However, for athletes, the primary outcome is performance. This difference necessitates the development of specific metrics to assess athlete performance within built environments. Future research should aim to establish these metrics, considering both traditional indicators like league rankings, Olympic medals and personal records, and potentially new metrics that accurately reflect the impact of built environments on athletic performance and health.

A major barrier to progress in this field is the lack of built environment subjects in traditional sports science curricula. This may limit awareness of the built environment’s impact on athletes’ performance. Further collaborations between sports scientists and scientists in urban design, parks, transportation, landscape architecture and environmental psychology can bridge this gap.
